# Genome-Wide Identification and Expression Analysis of eIF Family Genes from *Brassica rapa* in Response to TuMV Resistance

**DOI:** 10.3390/plants11172248

**Published:** 2022-08-30

**Authors:** Wenyue Huang, Shaoxing Wang, Shifan Zhang, Fei Li, Hui Zhang, Rifei Sun, Shujiang Zhang, Guoliang Li

**Affiliations:** 1 Institute of Vegetables and Flowers, Chinese Academy of Agricultural Sciences, Zhongguancun, Nandajie No. 12, Haidian District, Beijing 100081, China; 2 College of Horticulture, Vegetable Genetics and Breeding Laboratory, Anhui Agricultural University, Changjiang West Road, No. 130, Hefei 230036, China

**Keywords:** eIF gene family, *Brassica rapa*, TuMV, resistance, expression analysis

## Abstract

*Brassica rapa* is one of the most important leafy vegetables worldwide, and has a long history of cultivation. However, it has not been possible to completely control the damage of turnip mosaic virus (TuMV), a serious virus in *B. rapa*, to production. In this study, the genome-wide identification and expression detection of eIF family genes from *B. rapa* in response to TuMV resistance were analyzed, including the identification of eIF family genes, chromosomal distribution, three-dimensional (3D) structure and sequence logo analyses, and the expression characterization as well as differential metabolite analysis of eIF family genes in resistant/susceptible lines, which may further prove the whole-genome tripling (WGT) event in *B. rapa* evolution and provide evidence for the functional redundancy and functional loss of multicopy eIF genes in evolution. A qRT-PCR analysis revealed that the relative expressions of eIF genes in a susceptible line (80461) were higher than those in a resistant line (80124), which may prove that, when TuMV infects host plants, the eIF genes can combine with the virus mRNA 5′ end cap structure and promote the initiation of virus mRNA translation in the susceptible *B. rapa* line. In addition, the metabolite substances were detected, the differences in metabolites between disease-resistant and disease-susceptible plants were mainly manifested by altered compounds such as flavonoids, jasmonic acid, salicylic acid, ketones, esters, etc., which inferred that the different metabolite regulations of eIF family genes and reveal the resistance mechanisms of eIF genes against TuMV in brassica crops. This study may lay a new theoretical foundation for revealing eIF family gene resistance to TuMV in *B. rapa*, as well as advancing our understanding of virus–host interactions.

## 1. Introduction

In plants, the cap-binding protein eIF4E can interact with eIF4G to form the eIF4F complex, which can recognize the mRNA 5′ end cap structure and promote the initiation of mRNA translation [1]. eIF4G, the multi-subunit eIF3, and the 40S ribosomal subunit can form an initiation ternary complex, the 43S initiation complex, which facilitates the formation of the eIF4F complex. In all eukaryotic organisms, the eIF4E amino acids are highly conserved, which can interact with the mRNA 5′ cap structure [2]. What is similar is that eIF4G, which can interact with eIF4E, only recognizes a conserved YXXXXLΦ motif, and the eIF4F complex (eIF4G/eIF4E) forms to initiate the initiation of mRNA translation in plants [3,4]. Besides eIF4G, the eIF4E protein can combine different eIF proteins, namely 4E-BP1, p20, 4E-T, PGL-1, etc., which could present various functions, including mRNA transport from the nucleus to the cytoplasm, mRNA turnover, the initiation of mRNA translation, and mRNA translational repression [5]. In addition to plants, multiple eIF4E genes can be identified in many organisms, including fish, flies, birds, mammals, frogs, and nematodes [2,6]. Additionally, many eIF4E genes cannot interact with eIF4G and other proteins, which would provide evidence for the functional redundancy and functional loss of multi-copy eIF genes. Many studies have found that there is at least one eIF4E gene for the initiation of mRNA translation, and that other eIF genes could be responsible for abiotic stress, cell development, antiviral defense, eIF4E/iso4E promotes translation termination by binding to VPg protein and enhances eIF4E/iso4E binding to virus in vitro [7,8].

In plants, there are multi-copy eIF4E genes and isomers; eIF4E and its isomers are divided into three classes, which are based on the conservation of Tre43 and Tre56 in the amino acid sequences [2,9]. All of the three classes of eIF4E genes have different functions, including mRNA 5′ cap-binding, interaction with eIF4G or other proteins, and the regulation of the expression of various tissues, which may all provide evidence for the initiation of translation regulation [10]. As mentioned above, eIF4E can interact with eIF4G to form the eIF4F complex, and in plants, in a similar manner to eIF4E, eIF4G also has isomers, which have multi-copies to interact with eIF4E and eIF(iso)4E to form eIF4F and eIF(iso)4F complexes. The functions of several different eIF genes have been reported in different plant species (Table 1). With the further understanding of eIF family genes, more and more eIF genes will be discovered to be resistant to various viruses in plants.

In brassica crops, many genes that demonstrate resistance to TuMV have been mapped, such as *TuRB01* [35], *TuRB02* [35], *TuRB03* [36], *TuRB04* [37], *TuRB05* [38], *ConTR01* [17], *retr01* [17], *retr02* [39], *retr03* [40], the eIF(iso)4E gene has been shown to be strongly linked to the brassica recessive resistance genes *retr01*, *retr02*, and *trs* [15,17,39]. The recessive gene *retr02* was identified as an eIF(iso)4E gene, which is resistant to a TuMV C4 isolate in *B. rapa* [15,39], and because of a natural mutation in *retr02*, resulting in eIF(iso)4E mis-splicing and losing functionality, *retr02* showed broad-spectrum resistance to TuMV in *B. rapa*. In addition, there are many eIF(iso)4E copies, which may enable function redundancy, resulting in various resistances in *B. rapa* [16]. The yeast two-hybrid technique (Y2H) was used to identify some key amino acids in the eIF(iso)4E protein during interaction between eIF(iso)4E and TuMV VPg [15]. Furthermore, in our previous studies, three eIF(iso)4E copies and five eIF4E copies were identified in *B. rapa*, and through yeast two-hybrid technique (Y2H) as well as bimolecular fluorescent complimentary (BiFC) assays, it was observed that all of the eIF4E copies could not interact with TuMV VPg, but that eIF(iso)4E could [41,42]. Further research showed that some SNPs played an important role in the interaction between TuMV VPg and eIF(iso)4E copies, such as the SNPs A_154_C/T_556_C in TuMV VPg and the SNP T_106_C in eIF(iso)4E.c [41,42]. In addition to the *retr01* and *retr02* genes, the *retr03* gene was cloned in brassica crops, and the *retr03* gene was the eukaryotic translation initiation factor 2B-beta (*eIF2Bβ*), which could encode the eIF2B protein interacting with GTP-binding proteins to form the eIF2–GTP complex in the process of the initiation of translation [40]. More and more eIF family genes have participated in the TuMV resistance group in *B. rapa*; however, the genome-wide identification and expression analysis of the eIF supergene family from *B. rapa* in response to TuMV resistance have not been proceeded with.

In this study, the genome-wide identification and expression detection of the eIF gene family from *B. rapa* in response to TuMV resistance were analyzed, including the identification of eIF family genes, chromosomal distribution, the identification of physicochemical properties, phylogenetic analysis, evolutionary analysis, conserved analysis, and the expression characterization of eIF family genes in resistant/susceptible lines. The aim of the study is to select the eIF family genes with special domains, which may be associated with TuMV resistance in *Brassica rapa*. This study may provide a new theoretical foundation for revealing eIF family gene resistance to TuMV in *B. rapa*, as well as advancing our understanding of virus–host interactions.

## 2. Results

### 2.1. Identification of eIF Family Genes in B. rapa and Arabidopsis thaliana

Twenty-three and sixty-three eIF family genes were identified from *A. thaliana* and *B. rapa*, respectively. The detailed names and corresponding relationships of *A. thaliana* and *B. rapa* genes mentioned below are shown in Table S1. Including five eIF genes in *A. thaliana*, each eIF gene only has one homologous gene in *B. rapa*, these are AT5G57870, AT5G06000, AT3G13920, AT3G60240 and AT4G33250. However, other eIF genes in *A. thaliana* could correspond to two or more eIF gene copies in *B. rapa*. Five eIF genes in *A. thaliana* are homologous to two eIF genes in *B. rapa*, these are AT4G01290, AT2G39990, AT4G33250, AT1G13020, and AT1G29550. Twelve eIF genes in *A. thaliana* are homologous to three eIF genes in *B. rapa*, these are AT5G35620, AT2G24050, AT5G20920, AT5G27640, AT3G56150, AT3G57290, AT3G11400, AT3G19760, AT3G26400, AT4G18040, AT1G76810, and AT3G55620, which may verify the triple process in the *B. rapa* genome from the *A. thaliana* genome. In particular, the AT1G54270 gene in *A. thaliana* is homologous to eight eIF genes in *B. rapa*, and these are BraA01g037700.3C, BraA03g015000.3C, BraA03g036200.3C, BraA05g016480.3C, BraA05g033110.3C, BraA06g000760.3C, BraA08g001040.3C, and BraA08g001050.3C, which are the members of eukaryotic initiation factor 4A, and function in nucleic acid binding and ATP binding. Next, the AT4G11420 gene in *A. thaliana* is homologous to five eIF genes in *B. rapa*, and these are BraA02g028410.3C, BraA02g028420.3C, BraA03g027600.3C, BraA03g050990.3C, and BraA09g027030.3C, which could encode a subunit of eukaryotic initiation factor 3 and be required for the binding of mRNA to 40S ribosomal subunits.

### 2.2. Phylogenetic Analysis of eIF Family Genes Sequences

**Figure 1:**
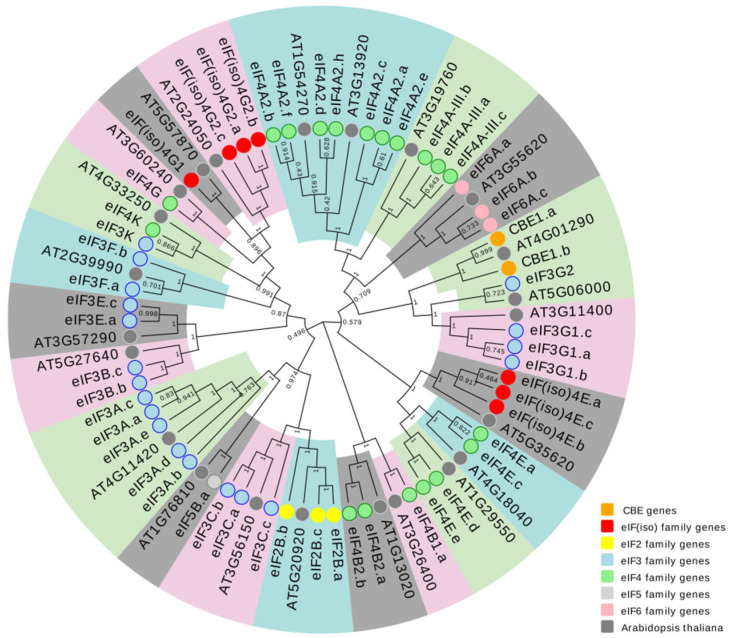
Phylogenetic analysis of 23 and 63 eIF family protein sequences from *A. thaliana* *and B. rapa*. Orange represents CBE genes, red represents class I (eIFiso family genes), yellow represents class II (eIF2 family genes), blue represents class III (eIF3 family genes), green represents class IV (eIF4 family genes), light grey represents class V (eIF5 family genes), pink represents class VI (eIF6 family genes), and grey represents *A. thaliana*.

To study the evolutionary relationships of eIF family genes, a total of 23 and 63 eIF protein sequences from *A. thaliana* *and B. rapa* were used to construct a phylogenetic tree (Figure 1). As the protein sequences of nine genes of *B. rapa* are too short and not highly conservative, they are not reflected in the figure. Combined with 23 eIF family genes in *Arabidopsis*, 54 eIF genes were mainly divided into six categories, namely, class I (eIFiso family genes), class II (eIF2 family genes), class III (eIF3 family genes), class IV (eIF4 family genes), class V (eIF5 family genes), and class VI (eIF6 family genes), the details can be found in Table S1. Class IV (eIF4 family genes) was the largest group, which included 24 genes (12 eIF4A, 5 eIF4B, 5 eIF4E, 1 eIF4K, and 1 eIF4G), and it was followed closely by class III, which included 21 eIF3 genes (5 eIF3A, 3 eIF3B, 3 eIF3C, 3 eIF3E, 2 eIF3F, 4 eIF3G, and 1 eIF3K). Class I was the eIFiso family genes, which included seven genes, and class II/V/VI had the same and the fewest genes, containing three genes each.

### 2.3. Chromosomal Distribution of eIF Family Genes in B. rapa

**Figure 2:**
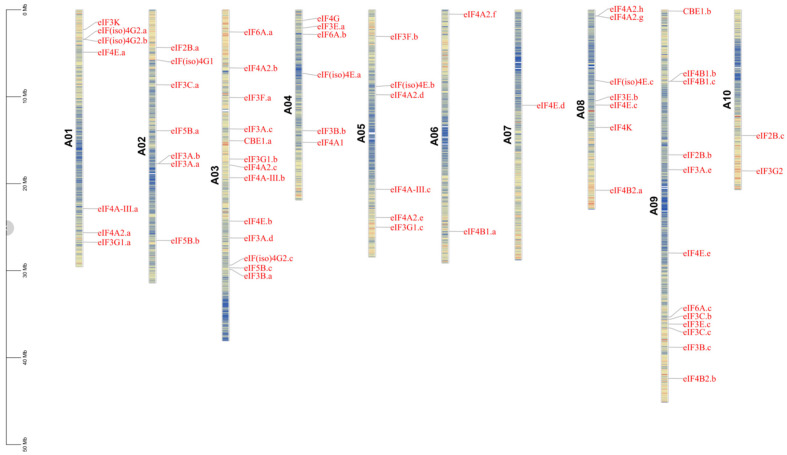
Chromosomal distribution of 63 eIF family genes from A01 to A10 of *Brassica rapa*.

A total of 63 eIF family genes were identified in *B. rapa*, and the eIF family genes were renamed according to their homology with *A. thaliana* genes from high to low (Table S1). The position of each gene on the chromosome is shown in Figure 2. There was only one gene (eIF4E.d) located on A07, and only two genes located on A06 (eIF4A2.f and eIF4B1.a) and A10 (eIF2B.c and eIF3G2) (Figure 2). On A04 and A05, there were six eIF genes each, and on A01, A02, and A08, there were seven eIF genes each (Figure 2). There were 13 eIF genes on A03, and these were eIF6A.a, eIF4A2.b, eIF3F.a, eIF3A.c, CBE1.a, eIF3G1.b, eIF4A2.c, eIF4A-III.b, eIF4E.b, eIF3A.d, eIF(iso)4G2.c, eIF5B.c, and eIF3B.a, which may be inferred to include more functions in A03. Next, there were 12 eIF genes located on A09. In addition, the functions of some eIF family genes have been verified, and the chromosomal distribution of the eIF family genes was consistent with previous studies [17,39].

### 2.4. Identification of the Physicochemical Properties of eIF Family Genes

A comparative genomic analysis showed that *B. rapa* and *A. thaliana* diverged from each other approximately 96 to 16 million years ago, that a whole-genome tripling (WGT) event occurred between 5.4 and 9 million years ago in *B. rapa*, and that there were three similar copes of genes with high-density distinct sub-genomes (MF1, MF2, and LF) [43]. In this study, there were 63 eIF family genes identified in *B. rapa*, including 25 genes located in the LF sub-genome, 24 genes located in the MF1 sub-genome, and 14 genes located in the MF2 sub-genome (Table S1). As we mentioned above, from 23 eIF genes in Arabidopsis, 12 of them are homologous to three eIF gene copies in *B. rapa*. Due to the WGT event, it was pondered whether the three eIF gene copies could be distributed on the MF1, MF2, and LF sub-genomes. In fact, they were not. There were only four eIF genes (AT5G35620, AT3G11400, AT3G19760, and AT4G18040) in *A. thaliana* that corresponded to three gene copies in *B. rapa* that were located on the MF1, MF2, and LF sub-genomes. Additionally, the corresponding three genes in *B. rapa* from the other eight eIF genes in *A. thaliana* were located on the LF/MF1 or LF/MF2 sub-genomes, at least one gene of the three copy genes was located on the LF sub-genome, which may imply that the LF sub-genome played an important role in the *B. rapa* genome. In addition, the 63 eIF family genes’ sequences were analyzed, and the cDNA sequences of the 63 eIF family genes were 219–3897 bp, while the protein sequences were 72–1298 aa. The large differences in the lengths of the protein sequences may suggest that the eIF family genes have different functions.

### 2.5. Evolutionary Analysis of eIF Family Genes in B. rapa

**Figure 3:**
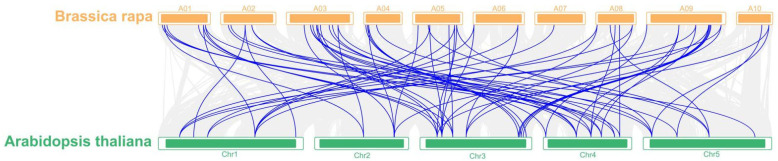
The collinearity analysis of eIF family genes between *B. rapa* and *A. thaliana*. A01 to A10 represent 10 chromosomes in *B. rapa*, Chr1 to Chr5 represent 5 chromosomes in *A. thaliana*.

The eIF family genes were further verified as going through genome tripling during *B. rapa* evolution based on whole-genome tripling (WGT), and to verify this hypothesis, the TBtools software was used to analyze the collinearity of sub-genomes in *B. rapa*. The results showed that the homologous gene pairs of eIF family genes were in the collinearity analysis section among different sub-genomes (Supplementary Figure S1). These results provide strong evidence for the hypothesis that eIF family genes were obtained through whole-genome tripling (WGT). In addition, the collinearity of the eIF family genes between *A. thaliana* and *B. rapa* genomes was also analyzed, and the results show that all of the 63 eIF family genes in *B. rapa* could match the homologous genes in *A. thaliana* through WGT during evolution (Figure 3). During the evolutionary selection pressure, there are many multi-copy genes and functional redundancy genes in *B. rapa*, most eIF family genes were strongly purified and selected, and only a few genes differentiated and produced new biological functions.

### 2.6. Conserved Analysis of the Gene Sequences and Gene Structures

**Figure 4:**
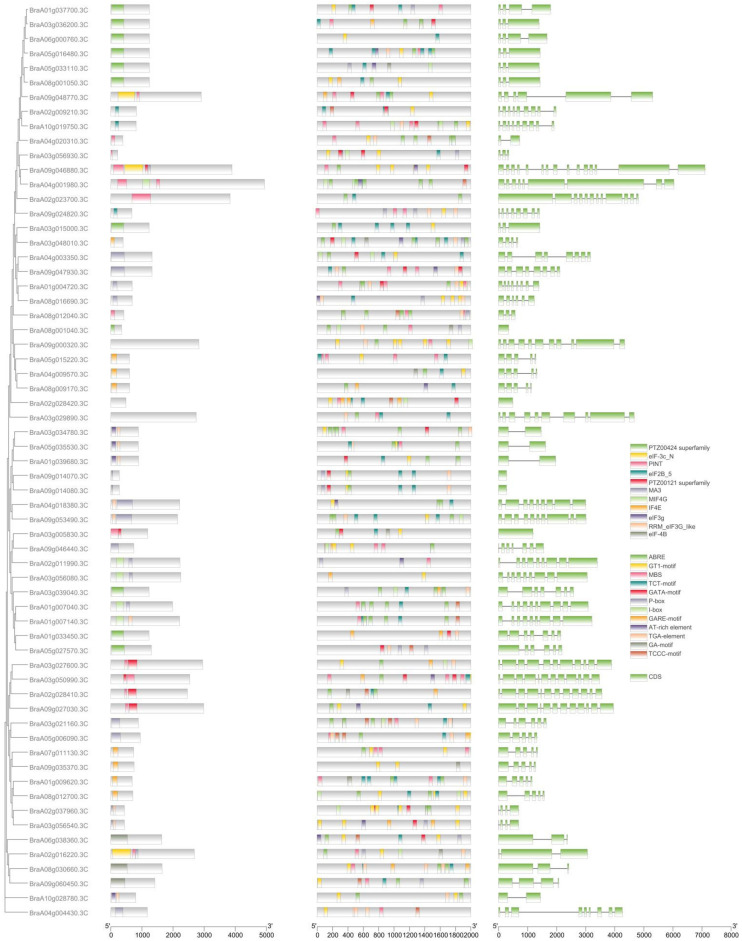
Conserved analysis of the 63 eIF family genes, including clustering analysis, amino acid sequences motif analysis, promoter *cis*-acting elements analysis, and the evaluation of exons and introns.

To understand the structural diversity and structural characteristics of the 63 eIF family genes in *B. rapa*, a motif analysis of the 63 eIF amino acid sequences was carried out by the MEME program. A total of 11 motifs were identified in the eIF genes, and the numbers and types of motifs in the eIF family genes were significantly different between groups, which indicated that the amino acid sequences and gene structures of the eIF family were not conserved and had functional differences. Only one eIF gene (BraA09g046880.3C) contained four motifs, and two eIF genes (BraA04g001980.3C and BraA02g016220.3C) contained three motifs. Most of the other eIF genes contained two motifs (Figure 4). In addition, to better study gene expression and transcriptional regulation, the *cis*-acting elements in the 63 eIF family genes’ promoter regions were analyzed by TBtools (Figure 4 and Table S3). Among the identified *cis*-acting elements, mainly stress- and hormone-related *cis*-acting elements were analyzed, and 203 were related to hormones, including gibberellin (GA), auxin (IAA), and abscisic acid (ABA). The light response elements (150) were the most common type, accounting for 30% of all of the identified *cis*-acting elements. A total of 498 *cis*-acting elements were related to stress, such as anaerobic conditions, drought, and low temperatures. Among these elements, the numbers of defense- and antioxidant-related reaction elements were higher. The results of these *cis*-acting elements suggested that eIF family genes may play an important role in hormone regulation and stress responses in *B. rapa*. In addition to the motifs and *cis*-acting elements analysis, the exons and introns of eIF family genes were analyzed and identified by TBtools (Figure 4). The number of exons of BraA09g046880.3C was the highest, containing 15 exons. A total of 12 eIF genes had 10 to 14 exons, 16 eIF genes had 6 to 9 exons, and 34 eIF genes had less than or equal to 5 exons. There were significant differences in the number of exons among different eIF family genes, and the genes were not relatively conserved in *B. rapa*.

### 2.7. Three-Dimensional (3D) Structure and Sequence Logo Analyses of eIF Proteins

**Figure 5:**
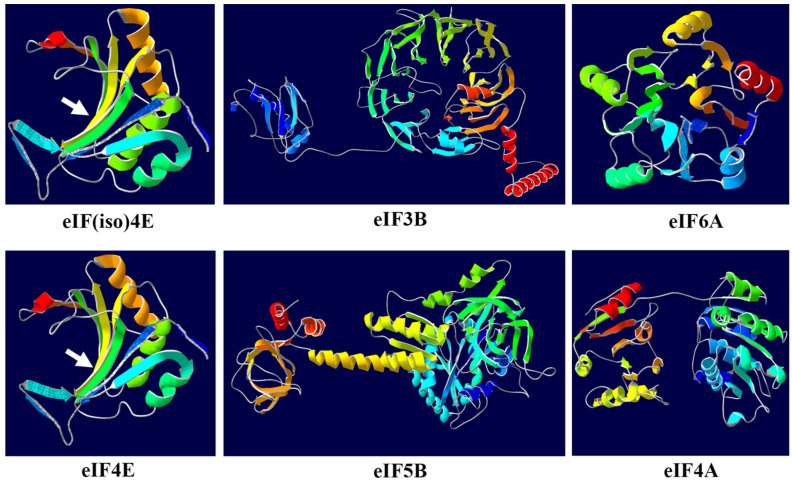
Three-dimensional (3D) structure analyses of the eIF(iso)4E, eIF3B, eIF6A, eIF4E, eIF5B, and eIF4A proteins of *B. rapa*. The white arrowheads of eIF(iso)4E and eIF4E were previously predicted to be key sites affecting TUMV resistance.

For confirming the functions of the protein structures, three-dimensional (3D) structural models of the eIF proteins were constructed, which were very different (Figure 5). The structure of the eIF(iso)4E protein is highly similar to that of the eIF4E protein, including eight *β*-strands, three *α*-helices, and three extended loops, which could form an inwardly depressed hydrophobic core structure that could be combined with the mRNA cap structure, playing an important role in resistance to TuMV in *B. rapa* [41,42]. The structure of the eIF3B protein contains RNA recognition motif (RRM), which consists of four strands and two helices arranged in an alpha/beta sandwich, with a third helix present during RNA binding [44]. The structure of the eIF6A protein also plays a role in the initiation of translation, acting as the translational GTPase that catalyzes the joining of the 40S and 60S subunits to form the 80S initiation complex with the initiator methionine-tRNA in the P-site base-paired to the start codon. Additionally, GTP binding and hydrolysis induce conformational changes in the enzyme that render it active for productive interactions with the ribosome [45]. The planform of the eIF5B protein structure is similar to a pentagon, which is endowed with special functions. The eIF5B protein may participate in various redox reactions, through the reversible oxidation of its active center dithiol to disulfide, and catalyzes dithiol–disulfide exchange reactions [46]. The structure of the eIF4A protein looks similar to a dumbbell, which encodes an ATP-dependent RNA helicase. The ATP-dependent RNA helicase is a subunit of the eIF4F complex involved in cap recognition, and is required for mRNA binding to the ribosome. In the current model of the initiation of translation, eIF4A unwinds RNA secondary structures in the 5′-UTR of mRNAs, which is necessary to allow for the efficient binding of the small ribosomal subunit in addition to subsequent scanning for the initiator codon [47]. Furthermore, in addition to the eIF proteins above, the structures of other eIF proteins also show various three-dimensional (3D) structures and functional characteristics (Supplementary Figure S2).

**Figure 6:**
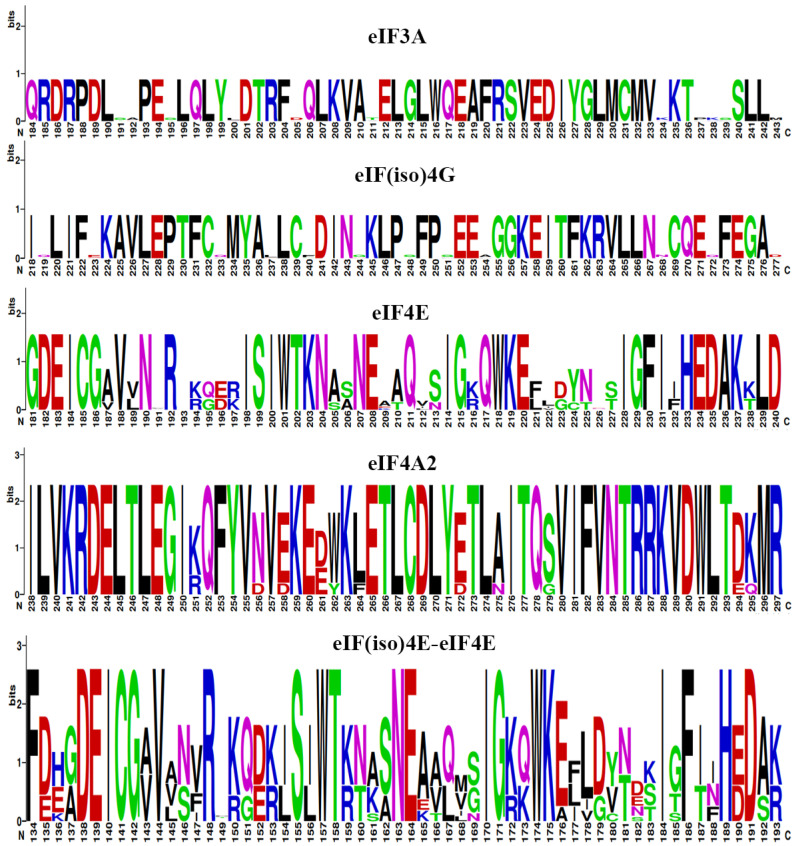
The sequence logo analyses of the eIF3A, eIF(iso)4G, eIF4E, eIF4A2, and eIF(iso)4E-eIF4E proteins of *B. rapa*.

Besides the three-dimensional (3D) structures, the sequence logos of the eIF family proteins were also analyzed (Figure 6). When the eIF3A.d sequence acted as the query, and the 184 to 243 amino acids of the eIF3A protein were analyzed, which encoded a conserved β-strand, it was found that the 11 sites, including 191/192/195, did not have obvious amino acid biases. When the eIF(iso)4G1 sequence acted as the query, and the 218 to 277 amino acids of the eIF(iso)4G protein were analyzed, which encoded a conserved α-helic, it was found that the 12 sites, including 219/223/233, did not have obvious amino acid biases. When the eIF4E.e sequence acted as the query, and the 181 to 240 amino acids of the eIF4E protein were analyzed, which encoded a conserved extended loop, it was found that the 187/224/225 sites had obvious amino acid biases, while the 191/226 sites did not. When the eIF4A2.b sequence acted as the query, and the 238 to 297 amino acids of the eIF4A2 protein were analyzed, which encoded a conserved α-helix, it was found that the 262/264/275/279 sites had obvious amino acid biases. From the 3D structure analysis, it can be seen that the structure of the eIF(iso)4E protein is highly similar to that of the eIF4E protein; therefore, the sequence logo of eIF(iso)4E combined with eIF4E was analyzed. When the eIF(iso)4E.a sequence acted as the query, and the 134 to 193 amino acids of the eIF(iso)4E–eIF4E proteins were analyzed, which encoded a conserved hydrophobic core structure, it was found that the 176 site has an obvious amino acid bias, while the 149/168 sites did not. In the co-evolution process of eIF genes, some amino acids were selected to turn into key amino acids for functions, and other amino acids were not selected to have amino acid biases. Analyzing the eIF family protein sequence logos would be helpful in screening the key amino acids for functions.

### 2.8. Expression Characterization of eIF Family Genes in Resistant/Susceptible Brassica rapa Lines

**Figure 7:**
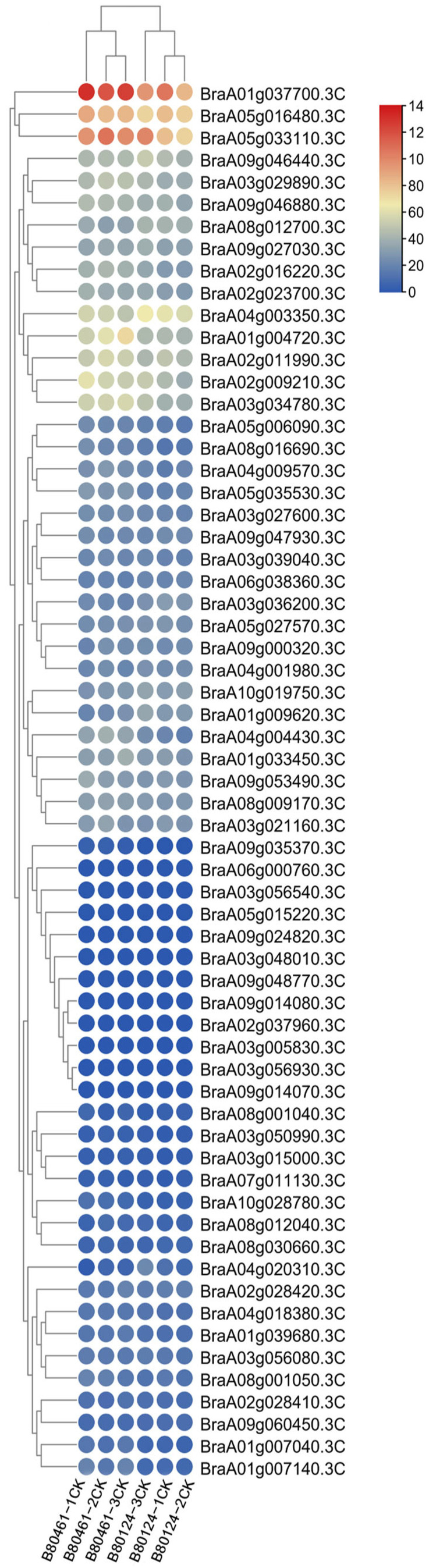
Expression characterization of the 63 eIF family genes between the resistant (80124CK) and susceptible (80461CK) *B. rapa* lines through transcriptome analysis.

**Figure 8:**
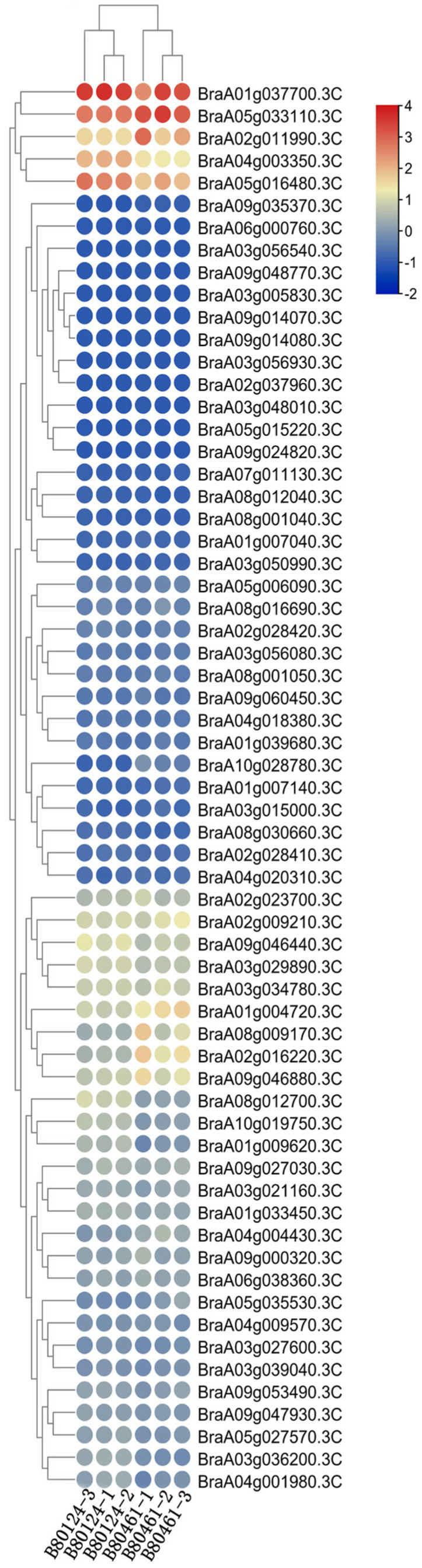
Expression characterization of the 63 eIF family genes between the inoculated–resistant (80124) and inoculated–susceptible (80461) *B. rapa* lines through transcriptome analysis.

To study the biological functions of eIF family genes, transcriptome analysis was performed to detect different expressions in the resistant (80124CK), inoculated–resistant (80124), susceptible (80461CK), and inoculated–susceptible (80461) *Brassica rapa* lines. Between 80124CK and 80124, some different expression genes were screened (Supplementary Figure S3A). Compared with 80124CK, the BraA02g011990.3C gene was a little upregulated in 80124, and the BraA05g033110.3C gene was downregulated in 80124. The expression of the four genes (BraA01g037700.3C, BraA05g016480.3C, BraA05g033110.3C, and BraA04g003350.3C) was much higher than that of the other 59 genes in 80124CK and 80124. However, between 80461CK and 80461, there were many different obvious expression genes (Supplementary Figure S3B). Compared with 80461CK, BraA05g033110.3C, BraA04g003350.3C, BraA08g009170.3C, BraA02g016220.3C, BraA09g046880.3C, and BraA02g011990.3C were upregulated in 80461, while BraA02g009210.3C, BraA03g034780.3C, BraA03g029890.3C, BraA09g046440.3C, and BraA05g016480.3C were downregulated in 80461 (Supplementary Figure S3B). The numbers of different expression genes were even greater between 80124CK and 80461CK (Figure 7). Compared with 80124CK, BraA01g037700.3C, BraA05g033110.3C, BraA09g046440.3C, BraA03g029890.3C, BraA01g004720.3C, BraA03g034780.3C, and BraA04g004430.3C were upregulated in 80461CK, while BraA08g012700.3C, BraA04g003350.3C, and BraA01g009620.3C were downregulated in 80461CK. Compared with 80124, there are more different expression genes in 80461 (Figure 8). The expression of BraA02g011990.3C, BraA05g033110.3C, BraA01g004720.3C, BraA08g009170.3C, BraA02g016220.3C, and BraA09g046880.3C was upregulated in 80461, while BraA01g037700.3C, BraA04g03350.3C, BraA05g016480.3C, BraA08g012700.3C, BraA10g019750.3C, and BraA01g009620.3C were downregulated in 80461 (Figure 8). After inoculating TuMV, the expression of many genes changed greatly, which may verify the notion that some eIF genes were the target genes of the TuMV effector for interaction with and resistance to TuMV in *B. rapa*.

### 2.9. qRT-PCR Analyses of eIF Family Genes in Resistant/Susceptible B. rapa Lines

**Figure 9:**
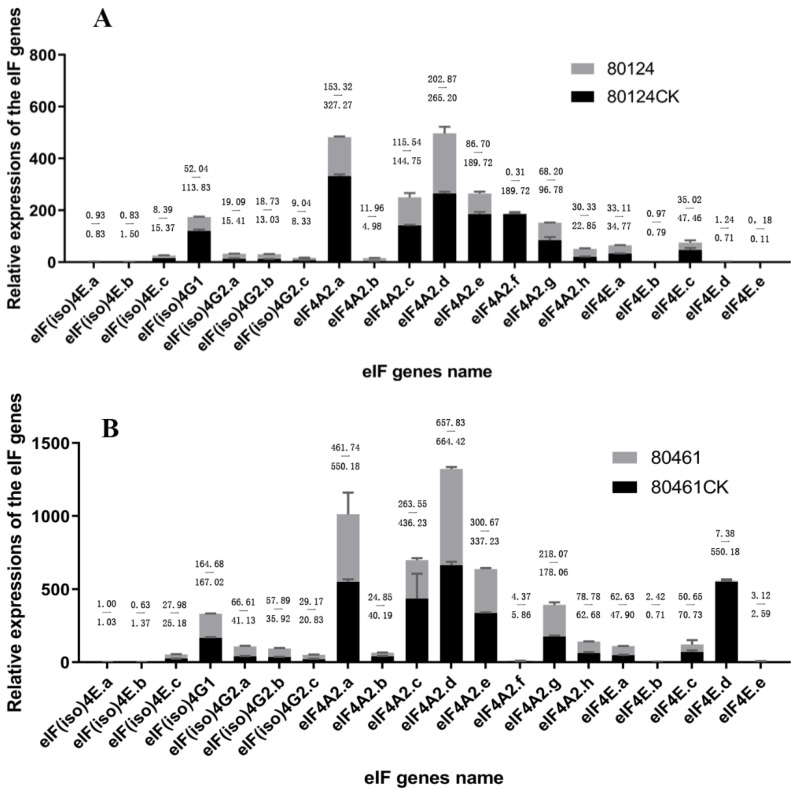
qRT-PCR analyses of eIF family genes in the resistant (80124CK) and inoculated–resistant (80124) (**A**), as well as the susceptible (80461CK) and inoculated–susceptible (80461) (**B**), *Brassica rapa* lines.

For a better understanding of the eIF genes’ functions for resistance against TuMV, the relative expressions of the eIF(iso)4E/eIF(iso)4G/eIF4A/eIF4E genes were detected in the resistant (80124CK), inoculated–resistant (80124), susceptible (80461CK), and inoculated–susceptible (80461) *Brassica rapa* lines (Figure 9). Compared with 80124CK, the expressions of the eIF genes saw little change or a slight decrease in 80124; in particular, the expression of eIF4A2.e was undetectable in 80124, which may be a negative regulation gene for TuMV (Figure 9A). Additionally, compared with 80461CK, the expressions of the eIF genes were slightly increased in 80461 (Figure 9B); in particular, the expression of eIF4E.d was undetectable in 80461, but it was very high in 80461CK, which may infer that TuMV could be a repressor against eIF4E.d expression in 80461 (Figure 9B). The expressions of eIF(iso)4E.a/eIF(iso)4E.b/eIF4E.b/eIF4E.e were very low or even undetectable, and in a previous study, eIF(iso)4E.b and eIF4E.b were proven to be pseudogenes [12]. Furthermore, the expression of eIF4A2.f was high in 80124CK, but it was undetectable in 80461CK, from which it may be speculated that the eIF4A2.f gene is an important gene for resistance against TuMV. On the contrary, the expression of eIF4E.d was high in 80461CK, but it was undetectable in 80124CK, which may suggest that the eIF4E.d gene interacts with TuMV and promotes the survival of TuMV in host plants. In addition, the relative expressions of eIF genes in 80461 and 80461CK were higher than those in 80124 and 80124CK, which may prove that, when TuMV infects host plants, the eIF genes can recognize the virus mRNA 5′ end cap structure and promote the initiation of virus mRNA translation in the susceptible *B. rapa* lines.

### 2.10. Differential Metabolites Analysis between the Susceptible and Resistant B. rapa Lines

For a better understanding of the functions of eIF family genes, metabolite substances were detected in the resistant (80124CK), inoculated–resistant (80124), susceptible (80461CK), and inoculated–susceptible (80461) *B. rapa* lines. There was only one metabolite (2,2′-Ethylidenebis(5-methylfuran), C_12_H_14_O_2_) downregulated between 80124 and 80124CK, and there were 24 differential metabolites between 80461 and 80461CK, including four upregulated metabolites and 20 downregulated metabolites (Table 2). It is worth noting that there are many volatile organic compounds (VOCs), such as butane dioic acid, allyl isothiocyanate, thiocyanic acid, etc., which are involved in the interactions among host plants (eIF genes), TuMV, and aphids. However, it was not clear which eIF gene could mediate the specific metabolites in *B. rapa*. In addition, there were 22 and 13 differential metabolites between 80124 and 80461 as well as 80124CK and 80461CK, respectively, including seven and four upregulated metabolites as well as fifteen and nine downregulated metabolites, respectively (Table S4). The metabolite substances were different between inoculated and non-inoculated *B. rapa* lines, indicating that there were significant differences in gene expression and metabolism. It is worthwhile to further explore the different metabolite regulations of eIF family genes and reveal the resistance mechanisms of eIF genes against TuMV in brassica crops.

## 3. Discussion

### 3.1. Evolution of eIF Family Genes

eIF family genes included many genes in brassica crops, such as 63 genes in *B. rapa*, 122 genes in *B. napus*, 69 genes in *B. oleracea*, and 132 genes in *B. juncea*, identified in the BRAD (http://39.100.233.196/#/ accessed on 1 December 2021). Previous studies have shown that the eIF4E and eIF4G genes could be available from sequenced Viridiplantae genomes, and proven that the eIF(iso)4E gene first appeared in flowering plants; however, the eIF(iso)4G gene appeared to expire earlier [9,48]. Additionally, the eIF4E-like and eIF(iso)4E-like genes appear to be sisters to each other, forming a monophyletic group, which in turn is a sister to the 4EHP-like lineage [48]. Many studies have found that there is at least one eIF4E gene for the initiation of mRNA translation, and many eIF4E genes could not interact with eIF4G and other proteins, which would provide evidence for the functional redundancy and functional loss of multi-copy eIF genes [7,8].

### 3.2. eIF Family Genes as Important Resistance Factors against Viruses in Plants

There are more and more eIF family genes that have been identified as resistance genes to viruses, and various variants have been found in eIF family genes, which would prove selectivity in the evolution process between plant resistance genes and viruses [48]. In particular, many natural mutants of eIF4E or eIF(iso)4E near the cap recognition pocket occurred in resistance potyviruses, such as *Pisum sativum*, *Lactuca sativa*, and *Capsicum* spp. [49,50,51]. In addition, many mutations could result in null or truncated eIF4E or eIF(iso)4E, which may be related to a decline in resistance, such as in *plum*, *Capsicum* spp., and *Arabidopsis* [29,52,53]. In addition to *Arabidopsis*, *Capsicum* spp., *Lactuca sativa*, *Pisum sativum*, and *plum*, there are many plants resistant against viruses due to eIF genes, such as *Phaseolus vulgaris* [54], *Solanum habrochaites* [33], *Hordeum vulgare* [55], *Citrullus lanatus* [22], *Prunus armeniaca* [28], *Solanum lycopersicum* [56], *Solanum tuberosum* [57], *Brassica rapa* [39], *Brassica juncea* [40], *Oryza sativa* [25], etc. There could be more and more eIF genes excavated from different plant species, including monocots and dicots, which could be responsible for resistance against viruses. How do eIF genes work to resist viruses in plants, and what are the resistance regulatory mechanisms between eIF genes and viruses? It was necessary to combine biochemical approaches, genetics, and biotechnology to answer this evolutionary conundrum, not only for the model plant, *A. thaliana*, but also for relevant brassica species.

### 3.3. Effects of the Key Amino Acids at 3D Structure on Resistance

Previous studies have summarized the natural mutations of eIF4E family members effects in some plants [8]. For example, in *B. rapa*, amino acid changes in *BraA.eIF4E.a*, *BraA.eIF4E.c*, *BraA.eIF(iso)4E.a*, and *BraA.eIF(iso)4E.c* influence its resistance [42]. In addition, many mutations could result in null or truncated eIF4E or eIF(iso)4E, which may be related to a decline in resistance, such as in *plum*, *Capsicum* spp., and *Arabidopsis* [29,52,53].

In this study, there are some special spatial structures, such as Pentagon, dumbbell and other shapes related to functions (Figure 5). The structure of the eIF4A protein looks similar to a dumbbell, which encodes an ATP-dependent RNA helicase. Amino acid changes at different locations have different effects. Mutation sites occurred at the key positions of the special structures, resulting in functional changes, associated with eIF4E-mediated resistance. The five amino acid variations of VPg of potato virus Y were independently correlated with the virulence of *pvr*^2^ resistance gene in pepper [19,20]. The G_107_R mutation in the 3D band of eIF(iso)4E protein affects VPg and cap binding and is associated with viral resistance in peas, tomatoes and peppers, while L_79_R, which is located in an external loop, could affect VPg, but not cap binding [33,51,58]. It is proved that the conserved structure of protein is related to virus resistance. It is important to analyze the three-dimensional (3D) structure, which would be helpful to understand how amino acid mutation affects function, or how the many mutant positions affect function.

## 4. Materials and Methods

### 4.1. Plant Materials and TuMV Inoculation

The resistant *B. rapa* line ‘80124’, and the susceptible *B. rapa* line ‘80461’, which were highly inbred lines, were planted in a greenhouse in Haidian, Beijing, China. Fifty plants of each of the two lines were inoculated with the virus. The plants were inoculated with TuMV in our previous study [59]. When the plants grew to four flat true leaves, the TuMV-C4 isolate was inoculated. After 25 d, the total RNA was extracted by the trizol method from fresh leaves of 80124CK, 80124 (inoculated–TuMV), 80461CK, and 80461 (inoculated–TuMV). In addition, the samples were immediately frozen in liquid nitrogen and stored at −80 °C.

### 4.2. Identification and Characterization of eIF Family Genes in A. thaliana and B. rapa

Twenty-three *A. thaliana* eIF family genes’ sequences were obtained from the *A. thaliana* database TAIR (Supplementary Table S1). The protein and genome sequences of *B. rapa* (V3.0) were downloaded from the Brassica Database (BRAD) (http://39.100.233.196/#/ accessed on 1 December 2021). HMMER 3.0 software was used to identify the 63 eIF genes from *B. rapa* with an E value threshold (E) of <10–40 [60]. The online Simple Modular Architecture Research Tool (SMART) was used to analyze the conserved domains of all of the candidate eIF protein sequences [61].

### 4.3. Phylogenetic Analyses of eIF Family Genes

To study the phylogenetic relationships among the eIF family proteins, multiple sequence alignments of 23 and 63 sequences from *A. thaliana* and *B. rapa* were carried out using the ClustalW function of MEGA 7.0 software, and the NJ method was used to compare the results. The bootstrap method setting value for the phylogenetic tree was 1000, and the default values were used for other parameters [62].

### 4.4. Chromosome Distribution of eIF Family Genes

According to an analysis of the *B. rapa* genome database, the physical locations of eIF family genes were determined. A collinearity analysis of protein sequences was performed using BLASTP and MCScanX software. TBtools was used to visualize the chromosome location and collinearity results [63].

### 4.5. Analysis of Conserved Motifs, Gene Structures, and Cis-Acting Elements

MEME 5.1.0 (http://meme-suite.org/tools/meme accessed on 3 December 2021) was used to analyze the conserved domains of eIF family genes, the maximum number of motifs was set to 11, and other parameters were set to default values. TBtools was used to analyze the members of the gene family by comparing the CDS sequences of eIF family genes [63]. CDS sequences and genome sequence data packages were downloaded from the BRAD. The 2000 bp upstream regions from the initiation codons (ATG) of the 63 eIF family genes were analyzed by PlantCARE software, and the *cis*-acting elements in the promoter were evaluated [64].

Sequence Logos and Three-Dimensional (3D) Structures of eIF Family Genes. Sequence logos of eIF family genes from *B. rapa* and *A. thaliana* were created by using WEBLOG [65]. Three-dimensional structures of eIF family genes were analyzed using Phyre2, as described previously [42].

### 4.6. Transcriptome Data Analysis, Quantitative Real-Time Polymerase Chain Reaction (qRT-PCR), and Statistical Analyses 

The fresh leaves of the resistant (80124CK), inoculated–resistant (80124), susceptible (80461CK), and inoculated–susceptible (80461) *B. rapa* lines were used to extract the total RNA. Total RNA of the test material was extracted by the TRlzol method, and the Agilent 2100 detected the quality of the resulting RNA. Qualified RNA samples were constructed as cDNA libraries, and the Agilent 2100 Bioanalyzer Bioanalyzer detected the fragment size and concentration of the libraries. Sequencing was performed by combined probe anchored polymerization (cPAS) technology to obtain a sequencing read length of 150 bp. A transcriptome analysis was conducted by MetWare (http://www.metware.cn/ accessed on 20 November 2021), as described in a previous study. Raw RNA-seq data were uploaded to the NCBI (SRA: PRJNA764554). A heat map was created by TBtools. In addition, an RNAprep Pure Kit (TIANGEN, Beijing, China) was used to extract the total RNA through qRT-PCR. The sequences of qRT-PCR primers are shown in Table S2. Actin was used as a control. An ABI 7500 real-time PCR system (Applied Biosystems, Waltham, MA, USA) was used to perform qRT-PCR experiments with three biological replicates. The relative expression levels of eIF family genes were calculated by the 2^−△△CT^ method. Microsoft Excel 2018 was used to analyze the experimental data. The Student’s t-test was used to calculate the *p*-values (* *p* < 0.05; ** *p* < 0.01).

## 5. Conclusions

According to the 23 eIF genes of A. thaliana in *B. rapa* by analogy to 63 eIF family genes, the eIF family gene classification was mainly divided into six categories. eIF genes underwent genomic triplication during the evolution of *B. rapa*, and most of one eIF gene of A. thaliana in *B. rapa* could be compared to three homologous genes. However, only a small number of genes diverged with new dual ecological functions. The promoters of the eIF gene family in *B. rapa* were also analyzed, and most of the cis elements were found to be associated with defense and antioxidant, suggesting that the eIF family may play an important role in keratin regulation and stress response in *B. rapa*.

In many plants, eIF4E and eIF4G were identified as the recessive resistance genes to the virus, and when the virus infected the host plants, the virus RNA could use eIF4E and eIF4G to initiate the translation of its own genome duplication in the host cell. This being the case, it was necessary to discuss the genome identification, evolution, and expression analysis of eIF family genes in plants, which would make clear the eIF family genes’ evolutionary relationships and be helpful in understanding the plant–virus interplay. Additionally, it was possible to reveal the impact of natural selection as a defense strategy against viruses [48].

## Figures and Tables

**Table 1:** The functions of eIF genes have been reported in different plant species.

Plant Species	Genes	Effect	References
*Arabidopsis thaliana*	eIF4E	Resistance to C1YVV	[11]
*Arabidopsis thaliana*	eIF(iso)4E	Resistance to TuMV/LMV	[12,13]
*Arabidopsis thaliana*	eIF4G	Resistance to CMV/TCV	[14]
*Brassica rapa*	eIF(iso)4E	Resistance to PPV/TuMV	[15,16,17]
*Capsicum* spp.	eIF(iso)4E	Resistance to PVMV/ChiVMV	[18]
*Capsicum* spp.	eIF4E	Resistance to PVY/TEV/PepMoV	[19,20,21]
*Citrullus lanatus*	eIF4E	Resistance to ZYMV	[22]
*Hordeum vulgare*	eIF4E	Resistance to BaMMV/BaYMV	[23]
*Lactuca sativa*	eIF4E	Resistance to LMY	[24]
*Oryza sativa*	eIF4G	Resistance to RTSV	[25]
*Phaseolus vulgaris*	eIF4E	Resistance to BCMV/C1YVV	[26]
*Pisum sativum*	eIF4E	Resistance to PsBMV/BYMV/C1YVV	[27]
*Prunus armeniaca*	eIF4E	Resistance to PPV	[28]
*Prunus domestica*	eIF(iso)4E	Resistance to PPV	[29]
*Ryza* spp.	eIF(iso)4G	Resistance to RYMV	[30,31,32]
*Solanum habrochaites*	eIF4E	Resistance to PVY/TEV	[33]
Wheat	eIF4G and eIF(iso)4G	Interact with the tobacco etch virus (TEV) 5′ cap; initiate TEV RNA translation	[34]
Wheat	eIF4G	Resistance to TEV	[34]

**Table 2:** The differential metabolites between the susceptible (80461CK) and inoculated–susceptible (80461) *B. rapa* lines.

Index	Compound	Formula	Class	Fold Change	Log_2_FC	Type
KMW0583	Trans-beta-Ionone	C_13_H_20_O	Terpene	0.4449	−1.1685	Down
KMW0504	Benzene, 1-ethenyl-4-methoxy	C_9_H_10_O	Aromatic	2.5819	1.3685	Up
KMW0499	4-Methylthiazole	C_4_H_5_NS	Heterocyclic compound	0.0067	−7.2183	Down
KMW0186	Octanal	C_8_H_16_O	Aldehyde	2.9778	1.5743	Up
KMW0361	Benzaldehyde, 4-ethyl	C_9_H_10_O	Aldehyde	0.4638	−1.1086	Down
KMW0110	Allyl Isothiocyanate	C_4_H_5_NS	Ester	0.0000	−16.8360	Down
KMW0152	Butane, 1-isothiocyanato	C_5_H_9_NS	Ester	0.0329	−4.9257	Down
QWM0007	Butane dioic acid, diethyl ester	C_8_H_14_O_4_	Ester	0.0373	−4.7433	Down
WMW0081	Cis-2-(2-Pentenyl) furan	C_9_H_12_O	Heterocyclic compound	0.4219	−1.2452	Down
XMW0133	6-Methyl-6-(5-methylfuran-2-yl) heptan-2-one	C_13_H_20_O_2_	Ketone	0.4896	−1.0303	Down
XMW0048	Phenol, 2-nitro-	C_6_H_5_NO_3_	Phenol	7.2955	2.8670	Up
XMW0376	10-Methyltricyclo [4.3.1.1(2,5)] undecan-10-ol	C_12_H_20_O	Alcohol	0.4898	−1.0297	Down
XMW0460	1,3-Cyclopentadiene, 5,5-dimethyl-1,2-Dipropyl	C_13_H_22_	Olefin	0.2946	−1.7634	Down
NMW0073	Bicyclo-2-ene-2-carboxaldehyde, 6,6-dimethyl	C_10_H_14_O	Terpene	0.4031	−1.3107	Down
NMW0135	Thiocyanic acid, phenylmethyl ester	C_8_H_7_NS	Sulfide	0.3163	−1.6605	Down
D27	Benzene, (2-isothiocyanatoethyl)	C_9_H_9_NS	Sulfide	0.0043	−7.8497	Down
D218	Cyclohexanecarboxylic acid	C_7_H_12_O_2_	Acid	0.0614	−4.0261	Down
XMW0811	Phenethyl isocyanate	C_9_H_9_NO	Ester	0.2005	−2.3183	Down
XMW0812	Benzene, (1-methyl-1-propylpentyl)	C_15_H_24_	Aromatic	0.3611	−1.4697	Down
XMW1102	Vinyl trans-cinnamate	C_11_H_10_O_2_	Ester	2.1582	1.1099	Up
XMW1245	(+)-2-Carene, 2-isopropenyl	C_13_H_20_	Olefin	0.4683	−1.0946	Down
XMW1459	Thiophene, 2-butyl-5-ethyl	C_10_H_16_S	Heterocyclic compound	0.4348	−1.2017	Down
XMW1481	5′-Hydroxy-2′,3′,4′-trimethylacetophenone	C_11_H_14_O_2_	Ketone	0.3290	−1.6037	Down
D378	1-Butene, 4-isothiocyanato	C_5_H_7_NS	Sulfide	0.0012	−9.6749	Down

Notes: Index, the metabolites’ IDs. Compounds, the metabolite substances’ names. Formula, the molecular formulae of the metabolite substances. Class, the categories of the metabolite substances. Fold Change, the changed folds of the metabolite substances between the susceptible (80461CK) and inoculated–susceptible (80461) *B. rapa* lines. Log_2_FC, the logarithms of the changed folds. Type, up- or downregulation of the metabolite substances between the susceptible (80461CK) and inoculated–susceptible (80461) *B. rapa* lines.

## Data Availability

The data presented in this study are openly available in the NCBI with the BioProject number PRJNA764554. (https://www.ncbi.nlm.nih.gov/search/all/?term=PRJNA764554, accessed on 21 August 2021).

## Supplementary Materials

The following supporting information can be downloaded at: https://www.mdpi.com/article/10.3390/plants11172248/s1, Figure S1. The collinearity analysis of eIF family genes in the three subgenomes of *Brassica rapa*. A1 to A10 represent 10 chromosomes in *B. rapa*. Figure S2. The sequence logos analysis of eIF3A, eIF(iso)4G, eIF4E, eIF4A2, and eIF(iso)4E-eIF4E proteins of *B. rapa*. Figure S3. Expression characterization of 63 eIF family genes between resistant (80124CK) and inoculated-resistant (80124) *B. rapa* lines (A), between susceptible (80461CK) and inoculated-susceptible (80461) *B. rapa* lines (B) by transcriptome analysis. Table S1. 23 and 63 eIF family genes were identified from *A. thaliana* and *B. rapa*, respectively. Table S2. Promoter cis-acting elements analysis of the 63 eIF family genes. Table S3. The sequences of qRT-PCR primers for eIF(iso)4E, eIF(iso)4G, eIF4A, and eIF4E genes. Table S4. The differential metabolites between 80124 and 80461, 80124CK and 80461CK *B. rapa* lines.

## Abbreviations

RTSV (rice tungro spherical virus), CMV (cucumber mosaic virus), TCV (turnip crinkle virus), PepMoV (pepper mottle virus), PsBMV (pea seedborne mosaic virus), BYMV (bean yellow mosaic virus), BCMV (bean common mosaic virus), BaYMV (barley yellow mosaic virus), BaMMV (barley mild mosaic virus), ZYMV (zucchini yellow mosaic virus), MWMV (Moroccan watermelon mosaic virus), CVYV (cucumber vein yellowing virus), PVMV (pepper vein mottling virus), and ChiVMV (chilli veinal mottle virus).
