# Supplementary figures and images for: Genome-Wide Identification and Expression Analysis of eIF Family Genes from Brassica rapa in Response to TuMV Resistance
Source: Plants (Basel). 2022 Aug 30;11(17):2248. doi: 10.3390/plants11172248 (PMC9460045; doi:10.3390/plants11172248)

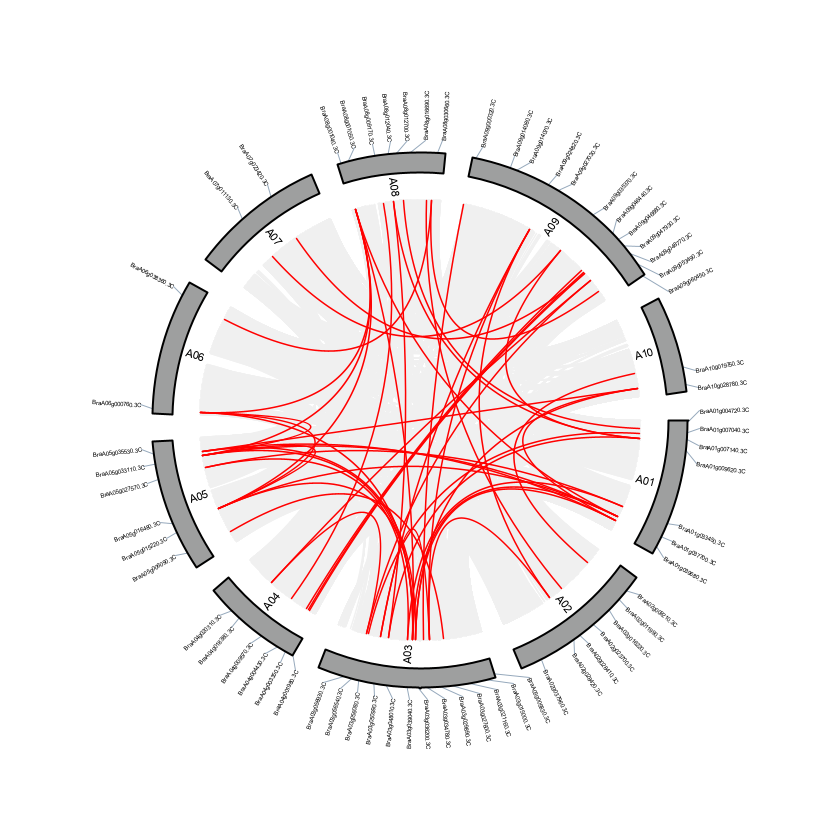

Supplement: Supplementary file 1 [file plants-11-02248-s001.zip › Supplementary Figure S1.png]

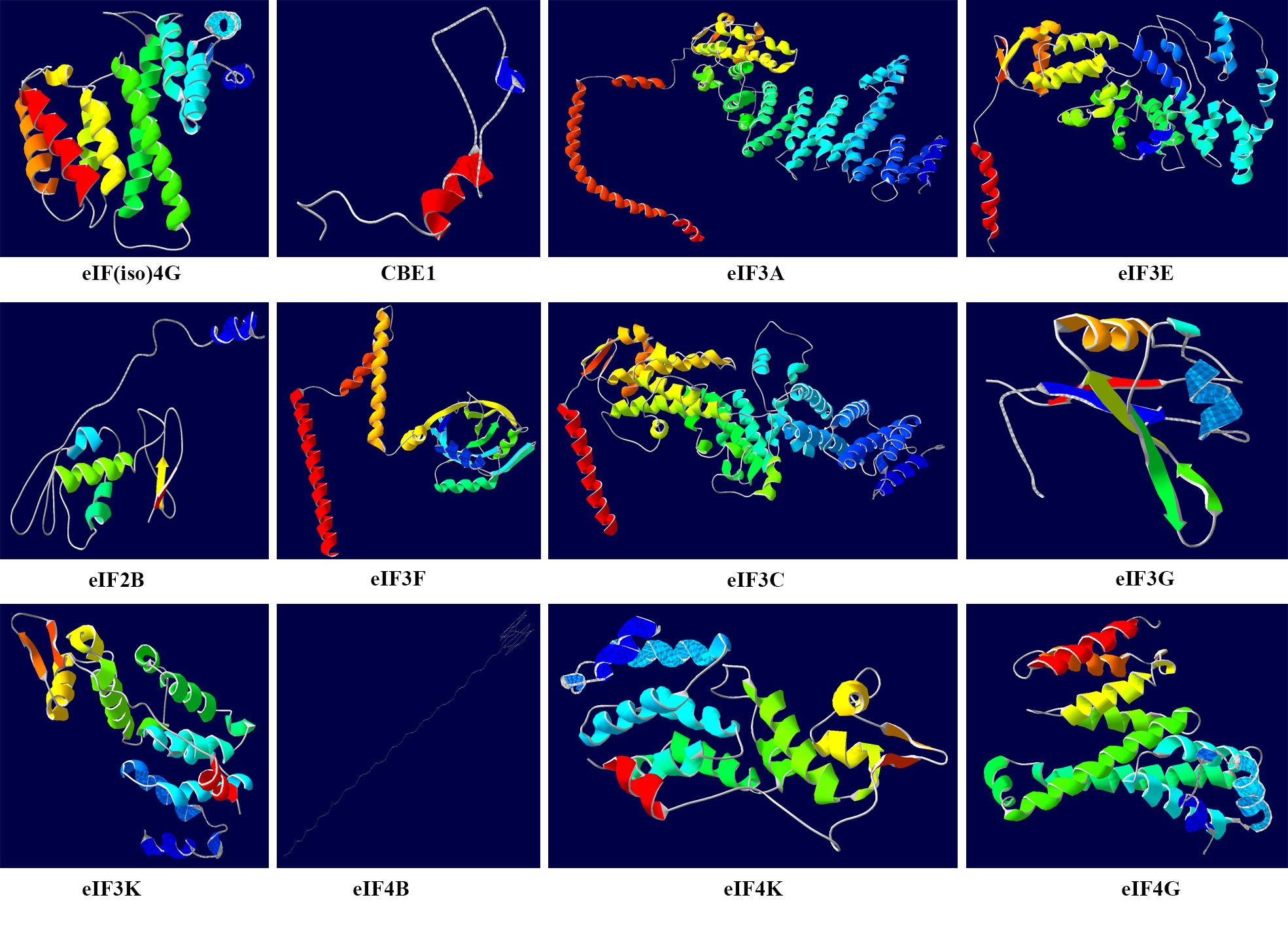

Supplement: Supplementary file 1 [file plants-11-02248-s001.zip › Supplementary Figure S2.tif]

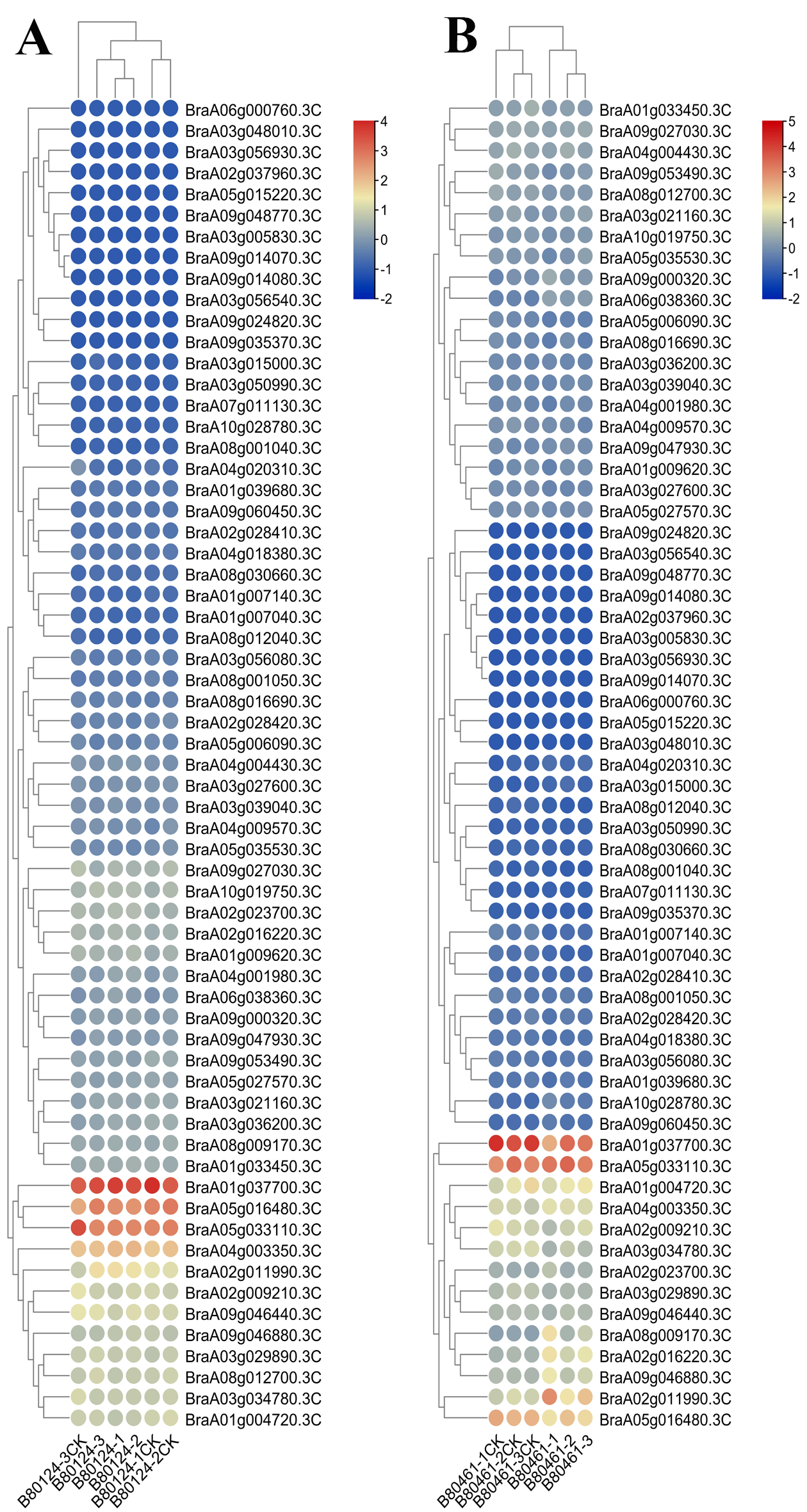

Supplement: Supplementary file 1 [file plants-11-02248-s001.zip › Supplementary Figure S3.tif]
